# Increased expression of Cyclin F in liver cancer predicts poor prognosis: A study based on TCGA database

**DOI:** 10.1097/MD.0000000000026623

**Published:** 2021-08-06

**Authors:** Yang Zelong, Yang Han, Guo Ting, Wang Yifei, He Kun, Hu Haoran, Chen Yong

**Affiliations:** a Department of Hepatobiliary Surgery, Xi Jing Hospital, Fourth Military Medical University, Xi’an, China; b School of Life Sciences, Central South University, Changsha, China; c Department of Obstetrics, West China Second University Hospital, Sichuan University, Chengdu, China; d Department of Neurology, Fourth Military Medical University, Xi’an, China.

**Keywords:** Cyclin F, GSEA, hepatocellular carcinoma, prognosis, TCGA

## Abstract

**Background:**

Cyclin F (CCNF) dysfunction has been implicated in various forms of cancer, offering a new avenue for understanding the pathogenic mechanisms underlying hepatocellular carcinoma (HCC). We aimed to evaluate the role of CCNF in HCC using publicly available data from The Cancer Genome Atlas (TCGA).

**Method:**

We used TCGA data and Gene Expression Omnibus (GEO) data to analyze the differential expression of CCNF between tumor and adjacent tissues and the relationship between CCNF and clinical characteristics. We compared prognosis of patients with HCC with high and low CCNF expression and constructed receiver operating characteristic (ROC) curves. In addition, we also explored the types of gene mutations in relevant groups and conducted Gene Set Enrichment Analysis (GSEA).

**Results:**

The expression of CCNF in liver cancer tissues was significantly increased compared with that in adjacent tissues, and patients with high CCNF expression had a worse prognosis than those with low CCNF expression. Patients with high CCNF expression also had more somatic mutations. High expression of CCNF hampers the prognosis independently. The GSEA showed that the "http://www.gsea-msigdb.org/gsea/msigdb/cards/BIOCARTA_WNT_PATHWAY" Wnt pathway, "http://www.gsea-msigdb.org/gsea/msigdb/cards/BIOCARTA_P53_PATHWAY" P53 pathway, "http://www.gsea-msigdb.org/gsea/msigdb/cards/HALLMARK_PI3K_AKT_MTOR_SIGNALING" PI3K/Akt/mTOR pathway, "http://www.gsea-msigdb.org/gsea/msigdb/cards/HALLMARK_NOTCH_SIGNALING" Notch pathway were enriched in patients with the high CCNF expression phenotype.

**Conclusion:**

High CCNF expression can be seen as an independent risk factor for poor survival in HCC. Its expression may serve as a target for the diagnosis and treatment of liver cancer.

## 1 Introduction

Liver cancer is a common malignant tumor that ranks sixth among new cancers in the world and is the fourth leading cause of cancer-related death.^[1]^ The high morbidity and high mortality are partly due to the insufficiency of current diagnostic techniques in identifying all early-stage liver cancer, and many liver cancers from common underlying diseases (such as chronic hepatitis B infection and alcoholic hepatitis). Although there are many ways to treat liver cancer,^[2]^ such as different liver surgery, radiofrequency ablation, radiotherapy and chemotherapy, immunotherapy, targeted therapy, but the overall therapeutic effect remains poor and the 5-year survival rate is less than 10%.^[2]^ It has become an urgent need to find more effective therapeutic and diagnostic targets and more accurate prognostic predictors.

There have been dozens of researches showed that Cyclin F (CCNF) is aberrantly expressed or mutated in some cancer types, however, whether or how the alteration in CCNF contributes to the tumorigenesis and tumor progression is not yet known.^[3]^ Studies have found that CCNF is in-frame fused in breast cancer^[4]^ and CCNF has recurrently mutated in about 20% of Burkitt lymphoma,^[5]^ which suggest that CCNF may play an operative role in cancers. There is a study that had shown that low CCNF expression is associated with poor prognosis of liver cancer,^[6]^ which is also the subject of our research, but our results differ from the previous study and we will discuss the reason in the discussion section. As an F-box protein, CCNF directly involves in the formation of Skp1-cullins-F-box complexes and regulates the protein ubiquitination and degradation. As a cyclin member, CCNF oscillates during the cell cycle, with protein levels peaking in the G2 phase. CCNF can regulate cell cycle transition processes, including centrosome homeostasis,^[7]^ genome stability maintenance,^[8]^ and DNA replication and repair.^[9]^ Despite its essential nature and status as the founding member of the 2-protein family, CCNF remains an orphan protein whose function in liver cancer is unidentified and controversial.

The abnormal expression of CCNF is related to various forms of cancer and certain neurological diseases, such as amyotrophic lateral sclerosis, frontotemporal dementia.^[10]^ In this study, we employed multiple bioinformatics analysis methods to inquire CCNF expression and elucidate the significance of abnormal CCNF expression in hepatocellular carcinoma (HCC) and its potential value for prognosis and diagnosis. The related functions and pathways were also investigated. Our results reveal that CCNF is noticeably up-regulated in liver cancer tissues and might impact the prognosis of HCC patients via regulating the Wnt pathway, the P53 pathway, the PI3K/Akt/mTOR pathway, and the Notch pathway.

## 2 Materials and methods

### 2.1 Data and clinical information mining and processing

The mRNA-sequencing data (level 3, Illumina HiSeq 2000 RNA Sequencing platform) of liver hepatocellular carcinoma (LIHC) and relevant clinical data were obtained from The Cancer Genome Atlas (TCGA) database portal (https://portal.gdc.cancer.gov/), which included 377 LIHC samples and 50 adjacent controls.^[11]^ GSE132037 data from Gene Expression Omnibus database were used as an external verification about the abnormal expression of CCNF in liver cancer, including 18 normal samples and 34 tumor samples.^[12]^ We divided the samples into high and low expression groups according to the median expression value of CCNF from the TCGA dataset. Overall survival (OS), disease-specific survival (DSS), disease-free interval (DFI), and progression-free interval (PFI) were analyzed between the high and low CCNF expression groups via Kaplan–Meier analysis using “survival” package in R software. We performed somatic gene mutation analysis with obtained somatic mutation data (level 4) in MAF format from TCGA using “maftools” package in R, and chosen the most significant different gene mutations to suggest the unidentified role of CCNF in somatic mutation in LIHC, which are deserved to be explored further. Gene ontology (GO) analysis and Kyoto Encyclopedia of Genes and Genomes (KEGG) analysis were conducted using “clusterProfiler” package in R. Correlation between CCNF and immune cells infiltration was studied through Cistrome web tool.^[13,14]^ This study was based on the public database mining without human or animal experiments, and the clinical data used had been anonymized, there was no ethical approval.

### 2.2 Gene set enrichment analysis

Gene Set Enrichment Analysis (GSEA) determines whether an a priori defined set of genes has statistically significant differences in expression under 2 different biological conditions.^[15]^ The GSEA was performed by the GSEA software from Broad Institute (http://software.broadinstitute.org/gsea/downloads.jsp). The *P* < .05 and FDR < 0.25 were set as the cutoff criterion. In the present study, to identify signaling pathways activated in liver cancer, we used GSEA to generate an ordered list of all genes according to their correlation with CCNF expression and then analyzed these genes via 4 databases: Hallmark, KEGG, Biocarta, and GO. The number of gene set permutations was 1000 for each analysis.

### 2.3 Statistical analysis

Spearman correlation analysis was utilized to explore the relationship between continuous variables. The differences in variables between groups were evaluated using Student *t* test and one-way ANOVA or Pearson chi-squared test. Univariate and multivariate Cox analyses were performed to screen out potential prognostic factors linked to OS. In addition, the area under the curve was ascertained, and the optimal cutoff value was derived after the creation of receiver operating characteristic (ROC) curves. R 3.6.3 and GraphPad Prism 8 were used to analyze statistics. *P* < .05 indicated a significant difference. Each statistical test was double-sided.

## 3 Results

### 3.1 The expression of CCNF in liver cancer tissues was significantly higher than that in adjacent normal tissues

**Figure 1:**
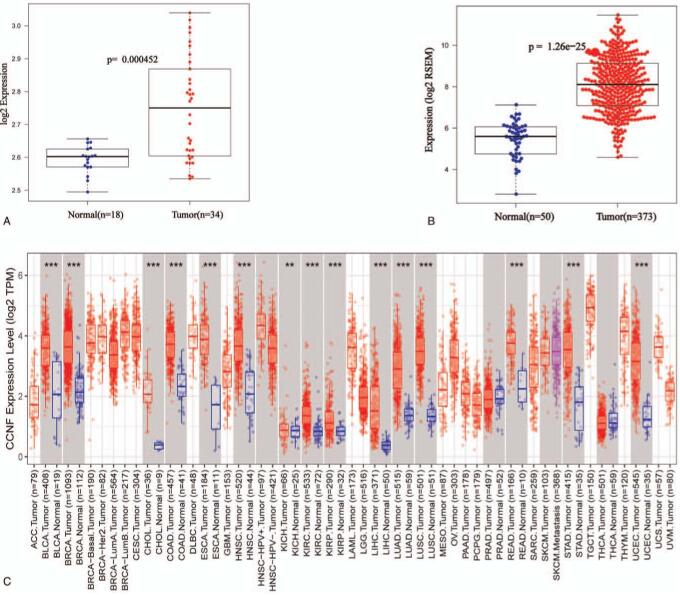
The expression of CCNF in liver cancer tissues is significantly upregulated compared with that in normal tissues. (A) In data obtained from GSE132037, the CCNF expression is significantly increased in HCC tissues compared to that in normal tissues (*P* = .000452). (B) In data obtained from TCGA, the CCNF expression is significantly increased in HCC tissues compared with that in normal tissues (*P* = 1.26e-25). (C) CCNF is highly expressed in multiple cancers. CCNF = Cyclin F, HCC = hepatocellular carcinoma, TCGA = The Cancer Genome Atlas.

The clinical data of 377 LIHC patients from TCGA were presented in Table 1. CCNF expression values were compared between tumor tissues and adjacent tissues, and the results showed that the expression of CCNF was significantly increased in tumor tissues compared to that in adjacent tissues (Fig. 1A, B). To evaluate the expression of CCNF in pan-cancer, we searched the TIMER database and found that the CCNF gene is highly expressed in various cancer types, including LIHC, breast invasive carcinoma (BRCA), and bladder urothelial carcinoma (BLCA) (Fig. 1C).

**Table 1:** Clinical characteristics of the included HCC patients from TCGA-LIHC.

Clinical characteristics	Number of patients
Sex	
Male	255
Female	122
Age	
<60	204
≥60	172
NA	1
Race	
White	187
Asian	161
Black or African American	17
American Indian or Alaska native	2
Not provided	10
Histologic grade	
G1	51
G2	180
G3	124
G4	13
Not provided	5
Pathologic stage	
Stage I	175
Stage II	87
Stage III	77
Stage IV	5
Not provided	24
Vital status	
Alive	286
Dead	91
Not provided	0
T	
T1	185
T2	95
T3	81
T4	13
TX	3
N	
N0	257
N1	4
NX	116
M	
M0	272
M1	4
MX	101

### 3.2 The somatic mutation rate of the CCNF high expression group was generally higher than that of the CCNF low expression group

**Figure 2:**
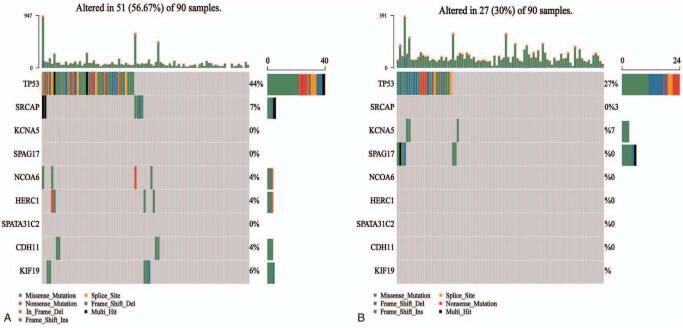
The somatic mutation rate of the CCNF high expression group is generally higher than that of the CCNF low expression group. (A) Fifty-one of 90 representative samples with high expression of CCNF have genetic mutations; the mutated genes are TP53, SRCAP, NCOA6, HERC1, CDH11, and KIF19. (B) Twenty-seven of 90 representative samples with low expression of CCNF have genetic mutations; the mutated genes are TP53, KCNAS, and SPAG17. The mutation types included missense mutation, splice site mutation, and nonsense mutation. CCNF = Cyclin F.

In the groups of cases with high and low expression of CCNF, gene mutations were detected (Fig. 2A, B). We extracted the top 90 samples from both the high and low expression groups and found that 51 of 90 samples with high expression of CCNF had genetic mutations (56.67%), the most significantly different mutated genes were TP53 (44%), SRCAP (7%), NCOA6 (4%), HERC1 (4%), CDH11 (4%), and KIF19 (6%). Twenty-seven of the 90 samples with low expression of CCNF had genetic mutations (30%), and the most significantly different mutated genes were TP53 (27%), KCNAS (3%), and SPAG17 (7%). The percentage here refers to the proportion of the number of individuals with the gene mutation to the number of 90 samples. The mutation types in both groups included missense mutation, splice site mutation, nonsense mutation, frame-shift deletion, in-frame mutation, in-frame deletion, multiple mutations, and frame-shift insertion. The differences in somatic mutations between the high expression and low expression groups indirectly confirm that CCNF may play a certain role in the somatic mutation of liver cancer. However, the reasons for the differences in these gene mutations are not yet known, and further exploration is needed.

### 3.3 High CCNF expression sub-group had a significantly poorer prognosis

**Figure 3:**
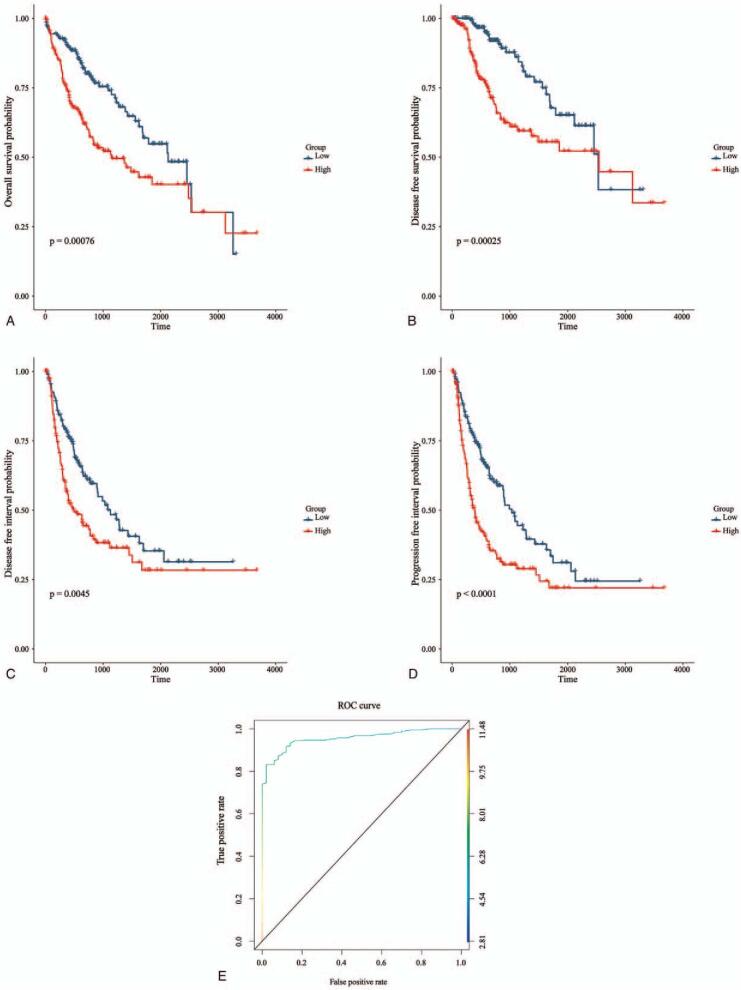
High CCNF expression is associated with a significantly poor prognosis. (A) Patients in the high CCNF group have a significantly worse OS than those in the low CCNF group (*P* < .0001). (B) Patients in the high CCNF group have a significantly worse DSS than those in the low CCNF group (*P* = .00025). (C) Patients in the high CCNF group have a significantly worse DFI than those in the low CCNF group (*P* = .00045). (D) Patients in the high CCNF group have a significantly worse PFI than those in the low CCNF group (*P* < .0001). (E) ROC curves of CCNF in predicting OS of LIHC. CCNF = Cyclin F, DFI = disease-free interval, DSS = disease-specific survival, LIHC = liver hepatocellular carcinoma, OS = overall survival, ROC = receiver operating characteristic curve.

To determine the prognostic impact of CCNF expression on the survival of HCC patients, Kaplan–Meier survival analysis was performed. As shown in Figure 3A to D, patients in the high CCNF group had significantly worse survival than those in the low CCNF group, the median survival time of OS in the low expression group in terms of OS, DSS, DFI, and PFI is 70.5, 84.4, 32.4, 30.4 months, respectively, and the median survival time of the high expression group is 45.7, 84.7, 16.8, 13.3 months, respectively, the hazard ratio (HR) is 1.7, 1.88, 1.32, 1.53, respectively. To assess the diagnostic value of CCNF, we generated a ROC curve using the expression data from HCC patients and healthy individuals (Fig. 3E). The area under the ROC curve (area under the curve) was 0.955496, which indicates that CCNF has a considerable diagnostic value.

### 3.4 CCNF expression hampered prognosis independently

**Figure 4:**
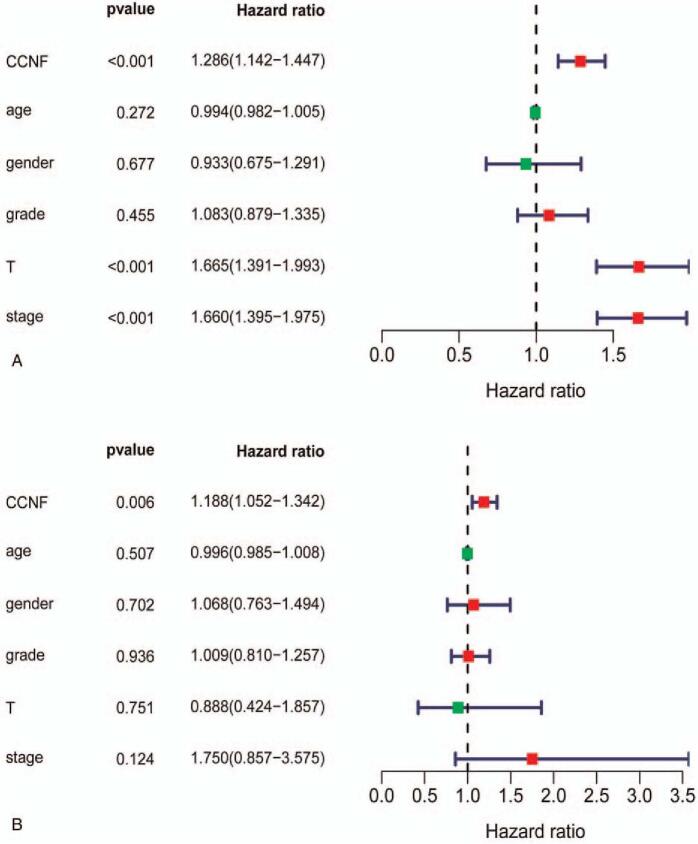
High expression of CCNF hampers OS independently. (A) The univariate Cox analysis shows that high CCNF (*P* < .001, HR = 1.286), tumor size (*P* < .001, HR = 1.665), and American Joint Committee on Cancer clinical stage (*P* < .001, HR = 1.66) were related to OS in LIHC. (B) The multivariate Cox analysis shows that high expression of CCNF (*P* = .006, HR = 1.188) is associated with OS. CCNF = Cyclin F, LIHC = liver hepatocellular carcinoma, OS = overall survival.

To evaluate the correlation between the high expression of CCNF and other factors linked to OS, we conducted univariate and multivariate Cox regression analyses. CCNF expression, tumor size, and clinical stage were shown to be responsible for the poor outcome of HCC patients in the univariate Cox analysis. The HR was 1.286, 1.665, and 1.6601, respectively. In the multivariate Cox analysis, only high expression of CCNF was associated with OS, with an HR of 1.188 (Fig. 4A, B).

### 3.5 Enrichment analysis of CCNF

**Figure 5:**
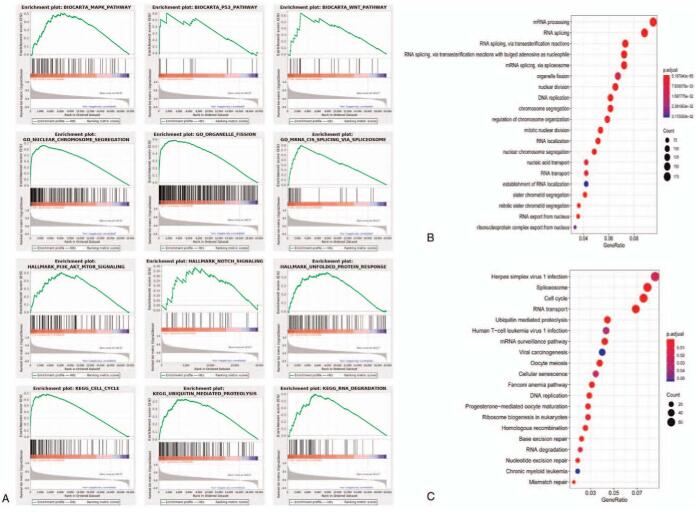
Enrichment plots from GSEA and function enrichment analyses in the high CCNF expression group. (A) GSEA results showing differential enrichment of genes related to the cell cycle pathway, the G2 pathway, the nuclear chromosome pathway, organelle fission, E2F targets, the G2M checkpoint, DNA replication, and base excision in HCC patients with high CCNF expression. (B) The top 10 significant GO terms. (C) The top 10 significant KEGG pathways. CCNF = Cyclin F, GSEA = Gene Set Enrichment Analysis, HCC = hepatocellular carcinoma, KEGG = Kyoto Encyclopedia of Genes and Genomes.

We defined the FDR < 0.25 and *P* < .05 as criteria for judging significance, and we used 4 gene sets, that is the biocarta gene sets, hallmark gene sets, GO gene sets, and KEGG gene sets to conduct GSEA analysis. There are MAPK pathway, Wnt pathway, P53 pathway, PI3K/Akt/mTOR pathway, and Notch pathway enriched in the high CCNF expression group, and biological processes such as unfolded protein response, cell cycle, and RNA degradation are also enriched in the gene sets with high CCNF expression (Fig. 5A). In addition, we conducted GO and KEGG analysis as the figure shows that there are some oncologic pathways or functions enriched in the high-expressed group, such as DNA replication, RNA splicing, and transport (Fig. 5B, C).

### 3.6 Website tool verification and protein–protein interaction network analyses

**Figure 6:**
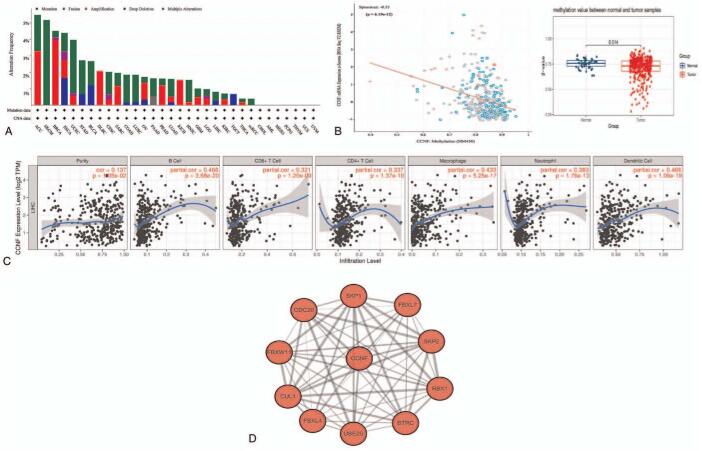
CCNF mutations exist in various cancers, immune infiltration cells are related to high CCNF expression in HCC. (A) Mutations of CCNF across cancers. (B) The degree of methylation of CCNF in adjacent tissues is higher than that in liver cancer. (C) The expression of CCNF is related to immune infiltration. (D) PPI analysis of CCNF and interacting proteins. CCNF = Cyclin F, HCC = hepatocellular carcinoma, PPI = protein–protein interaction network.

The cBioPortal for Cancer Genomics website (www.cbioportal.org) lists diverse cancers in which CCNF alterations (mutations, amplifications, or deletions) have been identified.^[16]^ We found that alterations in CCNF had potential great impacts on the occurrence and development of some tumors, such as adrenocortical carcinoma and melanoma (Fig. 6A). The methylation of the CCNF gene in adjacent normal tissues was higher than that in tumor tissues (*P* = .014), and the methylation of CCNF was negatively correlated with the expression of CCNF (Fig. 6B). Tumor tissues with high expression of CCNF showed high immune infiltration, and the expression of CCNF was partially positively correlated with tumor-infiltrating CD4+ T cells, CD8+ T cells, B cells, macrophages, neutrophils, and dendritic cells (Fig. 6C). Protein–protein interaction network was constructed by STRING database and visualized by Cytoscape 3.7.1. There were a total of 10 proteins interact with CCNF, of which 8 (SKP1, UBE2S, SKP2, CUL1, FBXL4, CDC20, FBXL7, and RBX1) have been confirmed by previous studies, and the other 2 proteins (BTRC and FBXW11) have been predicted to interact with CCNF (Fig. 6D).

## 4 Discussion

Cyclins are capable of interacting with CDKs to promote cell cycle progression and are frequently upregulated in human cancers. For instance, the level of cyclin B2 was higher in HCC than normal tissues and was associated with a poor prognosis in HCC patients.^[17]^ High cyclin D1 expression accompanied by low autophagic activity was correlated with liver tumor formation and poor survival in HCC patients.^[18]^ However, no CDKs interacting with CCNF have yet been found. Regardless, its role in tumors cannot be ignored. In 1994, Bai et al first reported CCNF as a founding member of the F box family, which is characterized by the presence of an F box motif.^[19]^ This discovery opened the door for research on CCNF in tumors. SiRNA-mediated depletion of CCNF in the G2 phase induces centrosomal and mitotic abnormalities, such as multipolar spindles and asymmetric, bipolar spindles with lagging chromosomes, and Skp1-cullins-F-box (CCNF)-mediated degradation of CP110 (a substrate of CNNF) is required for the fidelity of mitosis and genome integrity. CCNF can accelerate proliferation and increase in vitro tumorigenicity of ovarian cancer cells, and loss of CCNF results in severe mitotic defects, multipolar spindles, and supernumerary centrosomes, finally leading to the induction of apoptosis in ovarian cancer cells.^[20]^ The above abnormal phenotypes are seen consistently in different cancer models, indicating that CCNF has a general correlation with human cancer. In colon cancer, CCNF is highly expressed. Colon cancer patients with high CCNF expression show longer OS than those with low expression.^[21]^ This result is inconsistent with the conclusions we reached in this study, possibly because of the different tumor types. It is worth noting that in 5-FU-resistant colon cancer cell lines, the expression of CCNF is significantly upregulated, which is considered to be related to a drug-resistant phenotype. The expression of CCNF is elevated in oral cancer according to 1 report, and the logFC value was 2.3956.^[22]^ In regard to investigating the significance of the let 7 families of tumor suppressor microRNAs in malignant germ cell tumors, some researchers found that CCNF and let 7 expressions were negatively correlated, and CCNF was considered by the researchers to be an oncogene gene.^[23]^ Under metabolic stress conditions, CCNF promotes the degradation of RBPJ, and RBPJ can regulate IDH1, which is often mutated to an oncogenic form, IDH1-R132H, in cancers. Therefore, abrogation of CCNF expression facilitates IDH1-R132H-mediated tumorigenesis and metastasis in glioma; that is, CCNF may serve as a tumor suppressor in glioma.^[24]^

Unlike normal cells that transiently express small amounts of cyclin proteins at specific points in the cell cycle, many tumor cells have high constitutive levels of 1 or more cyclins.^[25]^ Here, we revealed that CCNF maybe can be used as a biomarker or a prognostic predictor for its elevation in HCC and negatively correlation with prognosis. Through univariate and multivariate Cox analyses, we found a possible relationship between CCNF expression and OS in HCC, and the ROC analysis confirmed the prognostic value of CCNF. Moreover, individuals with high expression of CCNF have more oncogenic mutations than those with low expression of CCNF. Through enrichment analysis, we found that the crucial biological signaling pathways enriched in the group with high CCNF expression are the Wnt pathway, P53 pathway, PI3K/Akt/mTOR pathway, Notch pathway, and MAPK pathway. However, the expression of CCNF and its potential prognostic impact on HCC were explored in 2013, and that study concluded that CCNF expression in HCC tissue is often decreased and that patients with low CCNF expression have a worse prognosis. In contrast, we found the opposite. These differences in results may be due to the following main reasons. Firstly, the former research was mainly based on clinical specimens and information collection and analysis, it included 245 HCC patients without verification in silicon, and our research was mainly based on the TCGA database. Secondly, the previous research mainly collected cases in China, and thus the patients mainly are Chinese, while our study collected data from the TCGA database, which includes data from multiple races. In addition, the previous study examined the patients of clinical stages I and II (111/245) and patients of clinical stages III and IV (134/245), but our research mainly focused on patients of clinical stages I and II (300/377), and the differences in our results may indicate that CCNF expression is different in different stages of liver cancer, suggesting its potential prognostic utility. We do not deny the conclusions of the previous studies. The conflicting results may have been caused by the listed differences. So further studies are needed.

Our analysis using online tools showed that mutations in CCNF influence the occurrence and development of some tumors; however, in 372 liver cancer patients, the mutation rate was only 0.81%, so CCNF mutation may not have a significant impact in liver cancer patients. However, some mutations in CCNF have been identified as “accelerants” in breast cancer,^[4]^ as well as in Burkitt lymphoma.^[5]^ In addition, methylation analysis of the CCNF gene showed that CCNF has a tendency to be highly expressed in liver cancer tissues.

Immune cells constitute an important element of tumor tissue, and accumulating evidence indicates their significance in predicting prognosis and therapeutic efficacy. Some scholars have classified the immune microenvironment of HCC into 3 distinct immunosubtypes: immune high, immune middle, and immune low. The immune high subtype is characterized by increased plasma cell and T cell infiltration, and the immune high subtype and B cell infiltration were identified as independent positive prognostic factors.^[26]^ Some scholars believe that high immune infiltration in tumor tissues prompts tumor cells to defensively upregulate various immune checkpoints to escape the immune system.^[27]^ Tumor-infiltrating lymphocytes are an independent predictor of sentinel lymph node status and survival in cancer. Although the correlation values were not statistically significant, CCNF was visibly related to all infiltrating immune cells, suggesting it may play a modest role in tumor growth and the maintenance of immune suppression.

In summary, by analyzing transcriptome and genome data, we found that CCNF is frequently upregulated in HCC. High CCNF expression unfavorably impacted the survival of HCC patients, and CCNF expression was significantly correlated with many factors that may affect prognosis, suggesting that CCNF plays a role in HCC initiation and progression. Collectively, our study revealed that high CCNF expression may be important for predicting the survival of patients suffering from HCC and that CCNF may serve as a target for the diagnosis and treatment of liver cancer.

## Author contributions

Yang Zelong and Hu Haoran designed research; Yang Zelong, Guo Ting, Wang Yifei and Hu Haoran performed research, Yang Zelong, He Kun and Yang Han analyzed data; and Yang Zelong, Yang Han and Hu Haoran wrote the paper. All authors read and approved the final manuscript.

**Data curation:** Yang Zelong, Guo Ting, Hu Haoran.

**Formal analysis:** Yang Zelong, Guo Ting, Yang Han.

**Funding acquisition:** Chen Yong.

**Investigation:** Yang Zelong, Wang Yifei, He Kun, Hu Haoran.

**Methodology:** Yang Zelong, Wang Yifei, He Kun, Guo Ting, Hu Haoran.

**Project administration:** Yang Zelong, He Kun.

**Resources:** Chen Yong. Hu Haoran.

**Software:** Yang Zelong, Guo Ting, He Kun, Yang Han.

**Supervision:** Chen Yong, Hu Haoran.

**Validation:** Chen Yong, Yang Zelong, Yang Han.

**Visualization:** Yang Zelong, Guo Ting, Yang Han.

**Writing – original draft:** Yang Zelong.

**Writing:** Yang Zelong, Yang Han, Hu Haoran.
